# Hypoxia Mediates Mutual Repression between microRNA-27a and PPARγ in the Pulmonary Vasculature

**DOI:** 10.1371/journal.pone.0079503

**Published:** 2013-11-14

**Authors:** Bum-Yong Kang, Kathy K. Park, David E. Green, Kaiser M. Bijli, Charles D. Searles, Roy L. Sutliff, C. Michael Hart

**Affiliations:** Departments of Medicine, Atlanta Veterans Affairs Medical Centers and Emory University, Atlanta, Georgia, United States of America; Vanderbilt University Medical Center, United States of America

## Abstract

Pulmonary hypertension (PH) is a serious disorder that causes significant morbidity and mortality. The pathogenesis of PH involves complex derangements in multiple pathways including reductions in peroxisome proliferator-activated receptor gamma (PPARγ). Hypoxia, a common PH stimulus, reduces PPARγ in experimental models. In contrast, activating PPARγ attenuates hypoxia-induced PH and endothelin 1 (ET-1) expression. To further explore mechanisms of hypoxia-induced PH and reductions in PPARγ, we examined the effects of hypoxia on selected microRNA (miRNA or miR) levels that might reduce PPARγ expression leading to increased ET-1 expression and PH. Our results demonstrate that exposure to hypoxia (10% O_2_) for 3-weeks increased levels of miR-27a and ET-1 in the lungs of C57BL/6 mice and reduced PPARγ levels. Hypoxia-induced increases in miR-27a were attenuated in mice treated with the PPARγ ligand, rosiglitazone (RSG, 10 mg/kg/d) by gavage for the final 10 d of exposure. In parallel studies, human pulmonary artery endothelial cells (HPAECs) were exposed to control (21% O_2_) or hypoxic (1% O_2_) conditions for 72 h. Hypoxia increased HPAEC proliferation, miR-27a and ET-1 expression, and reduced PPARγ expression. These alterations were attenuated by treatment with RSG (10 µM) during the last 24 h of hypoxia exposure. Overexpression of miR-27a or PPARγ knockdown increased HPAEC proliferation and ET-1 expression and decreased PPARγ levels, whereas these effects were reversed by miR-27a inhibition. Further, compared to lungs from littermate control mice, miR-27a levels were upregulated in lungs from endothelial-targeted PPARγ knockout (ePPARγ KO) mice. Knockdown of either SP1 or EGR1 was sufficient to significantly attenuate miR-27a expression in HPAECs. Collectively, these studies provide novel evidence that miR-27a and PPARγ mediate mutually repressive actions in hypoxic pulmonary vasculature and that targeting PPARγ may represent a novel therapeutic approach in PH to attenuate proliferative mediators that stimulate proliferation of pulmonary vascular cells.

## Introduction

Pulmonary hypertension (PH) is a devastating cardiopulmonary disorder defined by increases in pulmonary artery pressure and pulmonary vascular resistance that cause significant morbidity and mortality [1]–[3]. The complex pathogenesis of PH involves endothelial dysfunction, vasoconstriction, and abnormal proliferation of pulmonary vascular wall cells that lead to vascular remodeling and muscularization of small pulmonary vessels. Despite existing PH therapies designed to attenuate these derangements, the outcomes in PH remain poor indicating an urgent need for novel therapeutic strategies [2], [4]–[6].

Recent studies demonstrate that peroxisome proliferator-activated receptor gamma (PPARγ) regulates PH in several experimental models [7]–[12]. PPARγ is a ligand-activated transcription factor that belongs to the nuclear hormone receptor superfamily. PPARγ expression is reduced in the pulmonary vasculature of patients with severe PH [7]. Furthermore, smooth muscle cell (SMC)- and endothelial cell (EC)-targeted depletion of PPARγ caused spontaneous PH in mice [13], [14]. Therefore, reductions in PPARγ expression appear to be associated with PH and to promote PH pathogenesis [7], [12]–[14]. In contrast, activation of the PPARγ receptor with thiazolidinedione (TZD) ligands attenuates PH or vascular remodeling caused by monocrotaline [15] or hypoxia [8], [11] in rats, high fat diets in ApoE-deficient mice [9], or hypoxia in wild type mice [12]. Collectively these studies suggest that loss of PPARγ expression and function is associated with PH whereas pharmacological activation of PPARγ attenuates experimental PH.

The mechanisms by which PPARγ modulates the complex pathways involved in PH pathogenesis continue to be defined [8]–[12], [15], [16]. Reduced PPARγ signaling may contribute to PH pathogenesis through impaired production of vasoprotective signaling. For example, PPARγ participates in bone morphogenetic protein (BMP) signaling to regulate the expression of the vasodilator and antiproliferative mediator, apelin [17]. PPARγ also stimulates the expression of the anti-inflammatory adipocytokine, adiponectin, to attenuate PH in experimental models of insulin resistance [9]. These reports provide evidence that PPARγ stimulates the production vasoprotective mediators involved in PH. In contrast, PPARγ ligands also reduce the expression of proliferative mediators e.g. endothelin-1 (ET-1) [10] and Nox4 [16]. ET-1 is a potent endogenous vasoconstrictor that contributes to the pathogenesis of pulmonary arterial hypertension (PAH) [4], [18]. Plasma ET-1 concentrations are elevated in patients with PAH, and ET-1 mRNA and protein expression are increased in PAH endothelial cells and correlate with increased pulmonary vascular resistance [19]. Recent evidence demonstrates that PPARγ ligands attenuate PH in part by attenuating increases in ET-1 expression [10]. While endothelin receptor antagonists are employed in the clinical management of selected patients with PH, the simultaneous activation of ET-1-independent pathogenic pathways may limit the efficacy of inhibiting this single pathway. Indeed, the ability of PPARγ to simultaneously attenuate not only ET-1 signaling [10] but also additional proliferative pathways e.g. Nox4 [12], [16] and TSP-1 [20] provides additional rationale to further examine PPARγ in PH. Collectively, these studies suggest that loss of PPARγ signaling can simultaneously reduce the production of vasoprotective mediators while enhancing the production of proliferative mediators to promote PH pathogenesis.

Based on prior evidence that hypoxia reduces PPARγ expression in vitro and in vivo [11], [16], [21], the current study further examines mechanisms by which hypoxia reduces PPARγ. Evolving evidence indicates that dysregulation of microRNAs (miRNA or miR) contributes to PH pathogenesis [22]–[26] miRNAs are endogenous, noncoding, single-stranded RNAs of approximately 22 nucleotides that constitute a novel class of post-transcriptional gene regulators [27]. miRNAs negatively regulate the expression of their target genes through translational repression or mRNA degradation [27]. Recent studies have provided compelling evidence that miRNAs regulate pulmonary vascular remodeling by controlling EC and SMC differentiation and proliferation [22], [23], [25], [26], [28]–[30]. The potential role of miRNAs as master regulators in cell differentiation in physiologic and pathologic processes in the lung has recently been reviewed [31]–[33]. Taken together, these findings suggest that a better understanding of miRNAs in PH pathogenesis may provide new therapeutic opportunities in the management of human PH.

Although several miRNAs are aberrantly expressed in PH, the current study focused on miR-27a for several reasons. First, the 3′untranslated region (3′UTR) of PPARγ contains a binding site for miR-27, and miR-27 targets PPARγ mRNA in cardiomyocytes [34] and macrophages [35]. Second, the miR-27 gene family (miR-23a∼27a∼24-2 cluster) contributes to regulation of hypoxic responses including [36]–[38], cell cycle progression, proliferation, and hypertrophy [36]. Third, miR-27 is increased in the lungs of animals with PH [22] and in human pulmonary artery smooth muscle cell (HPASMC) isolated from patients with idiopathic PAH (IPAH) [30]. However the precise mRNA targets regulated by miR-27 and their contribution to the pathogenesis of PH have not been defined.

The findings in the current study provide novel evidence that hypoxia inhibits PPARγ expression and function through miRNA-mediated post-transcriptional mechanisms. Hypoxia increases miR-27a levels which reduce PPARγ expression and increase ET-1 expression and promote PH. On the other hand, activating the PPARγ receptor with pharmacological ligands attenuated hypoxia-induced increases in miR-27a. These findings provide evidence for a previously unrecognized mutually repressive relationship between PPARγ and miR-27a and indicate that strategies to maintain PPARγ expression and function in pulmonary vascular cells may provide therapeutic benefit in PH.

## Materials and Methods

### Ethical Statements

All animal studies were approved by the Institutional Animal Care and Use Committee of the Atlanta Veterans Affairs Medical Center.

### Reagents

3-(4,5-dimethylthiazol-2-yl)-2,5-diphenyltetrazolium bromide (MTT) was purchased from ATCC (Manassas, VA). ET-1, PPARγ, SP1, EGR1, CDK4, and beta-actin (ACTB) antibodies were obtained from Santa Cruz Biotechnology (Santa Cruz, CA). The following reagents were supplied by Sigma-Aldrich (St. Louis, MO): fetal bovine serum (FBS), dimethyl sulfoxide (DMSO), and methyl cellulose. Human scrambled siRNA, mimic-miR-27a, and anti-miR-27a were purchased from Qiagen (Valencia, CA). Human scrambled, SP1, and EGR1 Dicer-substrate 27-mer siRNA duplexes were purchased from IDT technologies (Coralville, Iowa). The PPARγ ligand, rosiglitazone, was obtained from Cayman Chemical (Ann Arbor, MI).

### Mouse Model of Chronic Hypoxia Exposure in vivo

C57BL/6 mice aged 8–12 weeks old were purchased from the Jackson Laboratory (Bar Harbor, ME). Endothelial- targeted PPARγ knockout (ePPARγ KO) mice were generated as previously reported [39]. Male mice aged 8–12 weeks old were exposed to 10% oxygen (hypoxia) or room air (control) for 3 weeks as reported [12]. Mice were housed socially and given unrestricted access to water and standard mouse chow. During the last 10 days of exposure to control or hypoxic conditions, selected animals were gavaged daily with rosiglitazone (10 mg/kg/day in 100 µL 0.5% methyl cellulose) or with an equivalent volume of vehicle alone as reported [12].

### In vitro Hypoxic Endothelial Cell Model

Human pulmonary artery endothelial cells (HPAECs, passages 3–7, Lonza, Walkersville, MD) were exposed to control conditions (21% O_2_) in a standard incubator or hypoxia (1% O_2_) in a Biospherix exposure chamber (Lacona, NY) for 72 hours as reported [10]. In separate experiments, HPAECs were examined following exposure to hypoxia for 0, 24, 48, and 72 h in a time-dependent manner. In selected studies, during the last 24 hours of normoxia or hypoxia exposure, HPAECs were treated with rosiglitazone (10 µM) or an equivalent volume of vehicle, and cell proliferation was measured using MTT assays as recently reported [10]. All manipulations of cells exposed to hypoxia were performed in a glove box which maintains the hypoxic environment to avoid effects of reoxygenation during sample processing.

### RNA Interference and HPAEC Transfection

Human PPARγ1 siRNA (NM_138712, Qiagen, Valencia, CA), siRNA duplexes (5′-CCCACTCCTTTGATATCAA-3′, target region 333–351) were designed with a BLOCK-it™ RNAi Designer (Invitrogen). siRNA targeted to a specific noncoding gene (5′-CCCUCCUAGUUUAUCACAAdTdT-3′) was employed as a scrambled RNA control. At 40–50% confluence, HPAECs were transfected with scrambled or PPARγ siRNA using GeneSilencer (Genlantis, San Diego, CA) transfection reagent according to manufacturer’s instructions. After transfection for 6 hours, the transfection media were replaced by endothelial cell growth medium (EGM) containing 10% FBS. HPAECs were cultured in fresh media for 72 hours. HPAEC lysates were then harvested and examined for PPARγ, ET-1, and miR-27a levels using qRT-PCR and Western blot assay. In separate experiments, HPAECs were transfected with scrambled or SP1 (5′-GGUGCAAACCAACAGAUUAUCACAA-3′, 3′-GUCCACGUUUGGUUGUCUAAUAGUGUU-5′) or EGR1 (5′-CCAUGGACAACUACCCUAAGCUGGA-3′, 3′-GUGGUACCUGUUGAUGGGAUUCGACCU-5′) RNAi duplex (10 nM) using RNAiMAX (Invitrogen) transfection reagent according to manufacturer’s instructions. After transfection, HPAECs were cultured for 72 hours. HPAEC lysates were then harvested and examined for SP1, EGR1, and miR-27a levels using qRT-PCR and Western blot assay. In selected studies, HPAEC proliferation was determined using MTT assays.

### Plasmid Construct, Luciferase Assays, and Site-directed Mutagenesis

The full 3′UTR of human PPARγ (NM_0138712), which contains the putative binding site for miR-27a, was amplified by RT-PCR with primers 5′-CATCAGCTCGAGCAGAGAGTCCTGAGCCACT-3′(Forward) and 5′-CGGATCGCGGCCGCACTATCAGCAATTTCATAATATGGT-3′(Reverse). After double digestion with *Xho*I and *Not*I restriction enzymes, the 236 bp amplicon was inserted into the *Xho*I and *Not*I restriction site of the psiCHECK2 vector (Promega), generating psiCHECK2-PPARγ-3′UTR, which was validated by DNA sequencing analysis. Positions 3–6 of the seed match were mutated by the QuikChange Site-Directed Mutagenesis Kit (Stratagene), termed psiCHECK2-PPARγ-3′UTR^mut^ (mut-3′UTR) using RT-PCR with primers 5′- ATTCTGAGGGAAAATCTGACACCTAAGAAATTT**ACACACAA**AAAGCATTTTAAAAAGAAAAGGTTTTAGAATAT-3′(Forward) and 5′- ATATTCTAAAACCT TTTCTTTTTAAAATGCTTT**TTGTGTGT**AAATTTCTTAGGTGTCAGATTTTCCCTCAGAAT-3′(Reverese), and confirmed by DNA sequencing analysis (Bold letters denote the miR-27a seed match sequence, with the mutated bases of the miR-27a seed match underlined.). HPAECs were plated in a 24-well plate (1×10^4^ cells/well), incubated for 24 h, washed with PBS, and then fresh growth medium was added before addition of transfection complexes. For luciferase assays, 100 ng of psiCHECK2-PPARγ construct, or mut-3′UTR construct, or psiCHECK2 vector with or without 30 nM of miR-27a mimic or scrambled miRNA were transiently cotransfected into HPAECs using GeneSilencer transfection reagent according to manufacturer’s instructions. After co-transfection for 6 hours, the transfection media were replaced by EGM containing 10% FBS. HPAECs were cultured in fresh media for 72 hours. HPAEC lysates were then harvested and analyzed for *Renilla* and firefly luciferase activity using the Dual Luciferase Reporter Assay System (Promega), and luciferase activity was measured using a Luminometer (PerkinElmer). Relative light units (RLU) were normalized to firefly luciferase activity.

### miR-27a Downregulation and Overexpression

To confirm the role of miR-27a in alterations in PPARγ expression, HPAECs (passages 3–7) were transfected, using GeneSilencer siRNA transfection reagent (San Diego, CA), with anti-miR-27a (25–50 nM) or an equivalent amount of anti-miR negative control for miRNA downregulation, or mimic miR-27a (30–50 nM) or scrambled siRNA for miRNA overexpression (Qiagen). After transfection for 6 hours, the transfection media were replaced by EGM containing 10% FBS. HPAECs were then cultured in fresh media for 72 hours and exposed to normoxia or hypoxia. Alterations in miR-27a, ET-1, and PPARγ levels were examined using qRT-PCR and Western blotting.

### miRNA and mRNA Quantitative Real-time Polymerase Chain Reaction (qRT-PCR) Analysis

To measure miR-27a, PPARγ, and ET-1 levels in HPAECs or mouse lungs, small RNAs (<200 nt) and large RNAs (>200 nt) were isolated using the mirVana kit (Invitrogen). The levels of miR-27a expression were analyzed by qRT-PCR using Qiagen miRNA primer assay (Qiagen) according to the manufacturer’s instructions. RNU6B (miRNA) was used as an exogenous control. PPARγ and ET-1 mRNA levels in the same sample were determined and quantified using specific mRNA primers as previously described [10]. 9S mRNA (mRNA) was used as an exogenous control.

### Western Blot Analysis

After treatment with normoxia or hypoxia ± rosiglitazone, mouse lung or HPAEC protein lysates were subjected to Western blot analysis as reported [12]. Primary antibodies included ET-1, PPARγ, CDK4, and ACTB. Proteins were visualized using a peroxidase-coupled anti-goat, anti-rabbit, or anti–mouse IgG in the presence of LumiGlo reagent (Thermo Scientific). Bands were identified by chemiluminescence, quantified by laser densitometry (Bio-Rad), and normalized to CDK4 or ACTB levels within the same lane.

### Statistical Analysis

For all measurements, data are presented as mean ± standard error of the mean (SE). All data were analyzed using analysis of variance (ANOVA). Post hoc analysis using the Student Neuman Keuls test was employed to detect differences between specific groups. In studies comparing only two experimental groups, data were analyzed with Student’s t-test to determine the significance of treatment effects. The level of statistical significance was taken as p<0.05.

Statistical analyses were carried out using software GraphPad Prism, Version 5.0 (LaJolla, CA).

## Results

### Hypoxia Increases miR-27a in in vivo and in vitro

**Figure 1:**
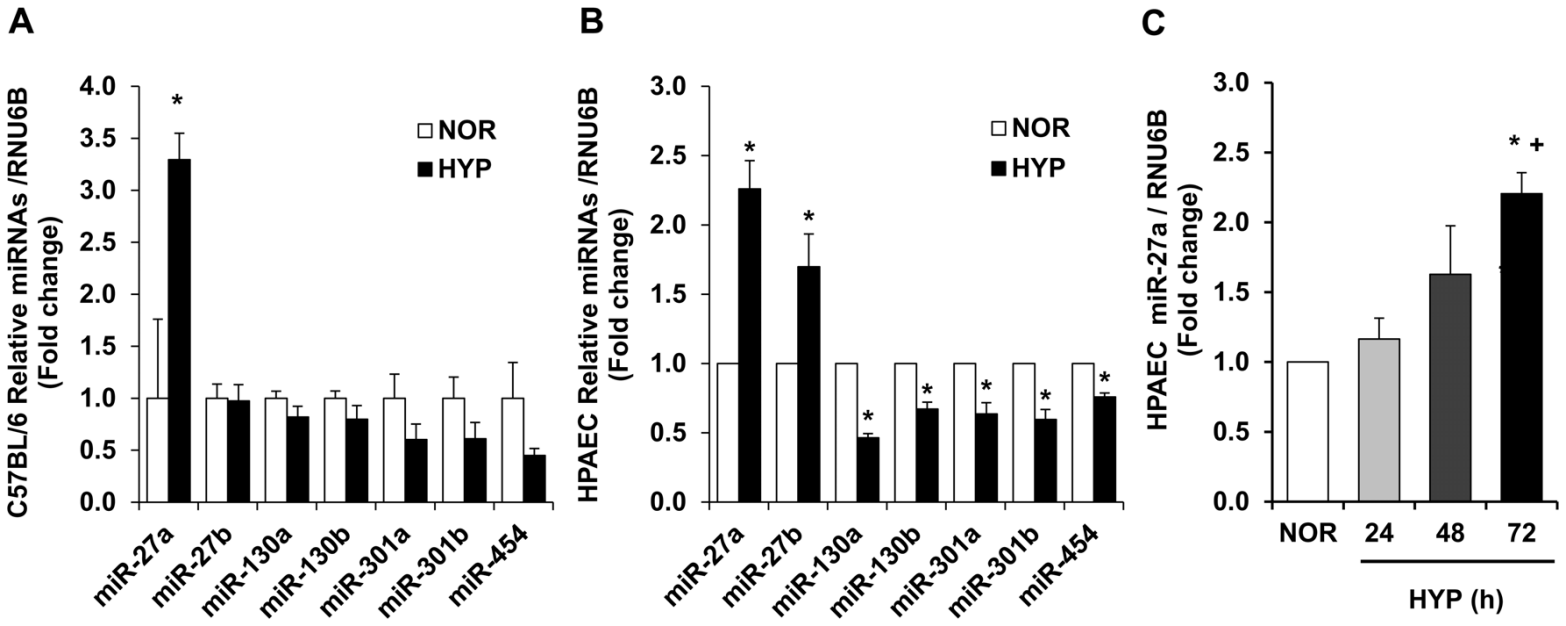
microRNA levels in hypoxia-exposed C57BL/6 mice and HPAECs. **A.** C57BL/6 mice were exposed to normoxia (NOR, 21% O_2_) or hypoxia (HYP, 10% O_2_) for 3-wks. qRT-PCR was performed on lung tissue for selected miRNAs predicted to regulate PPARγ expression. miRNA levels are expressed relatives to RNU6B and normalized to control values. *p<0.05 vs. NOR, n = 6–7. **B.** HPAEC were exposed to NOR or HYP (1% O_2_) in vitro for 72 h. n = 3, * p<0.05 vs. NOR. C. HPAECs were also examined following exposure to hypoxia for 0, 24, 48, or 72 h. HPAEC miRNA was isolated and subjected to qRT-PCR analysis along with RNU6B. Each bar represents the mean ± SE miR-27a relative to RNU6B expressed as fold change vs. SCR. n = 3, *p<0.05 vs. NOR. ^+^p<0.05 vs. 24 h.

To examine potential miRNAs that regulate PPARγ, a bioinformatics approach using multiple prediction algorithms (miRBase, PicTar, and TargetScan v6.1) was employed to identify binding sites for miRNAs in the 3′UTR of PPARγ. This analysis indicated miR-27a/b, miR-130a/b, miR-301a/b, and miR-454 as potential regulators of PPARγ. Samples from hypoxia-exposed mouse lungs or HPAECs were employed to screen for hypoxia-induced alterations in these miRNAs. As illustrated in Figure 1A, of the miRNAs that were examined and predicted to regulate PPARγ, hypoxia selectively increased miR-27a in mouse lung. As illustrated in Figure 1B, compared to control conditions, hypoxia induced a roughly 2-fold increase in levels of miR-27a and miR-27b in HPAECs in vitro that occurred in a time-dependent manner (Figure 1C). In contrast, hypoxia reduced levels of other miRNAs predicted to regulate PPARγ. Because increased levels of miR-27a are predicted to reduce PPARγ in mouse lung and in HPAECs, we focused on increased miR-27a levels as a putative mechanism for previously reported reductions in PPARγ [12], [16].

### PPARγ is a Target of miR-27a

**Figure 2:**
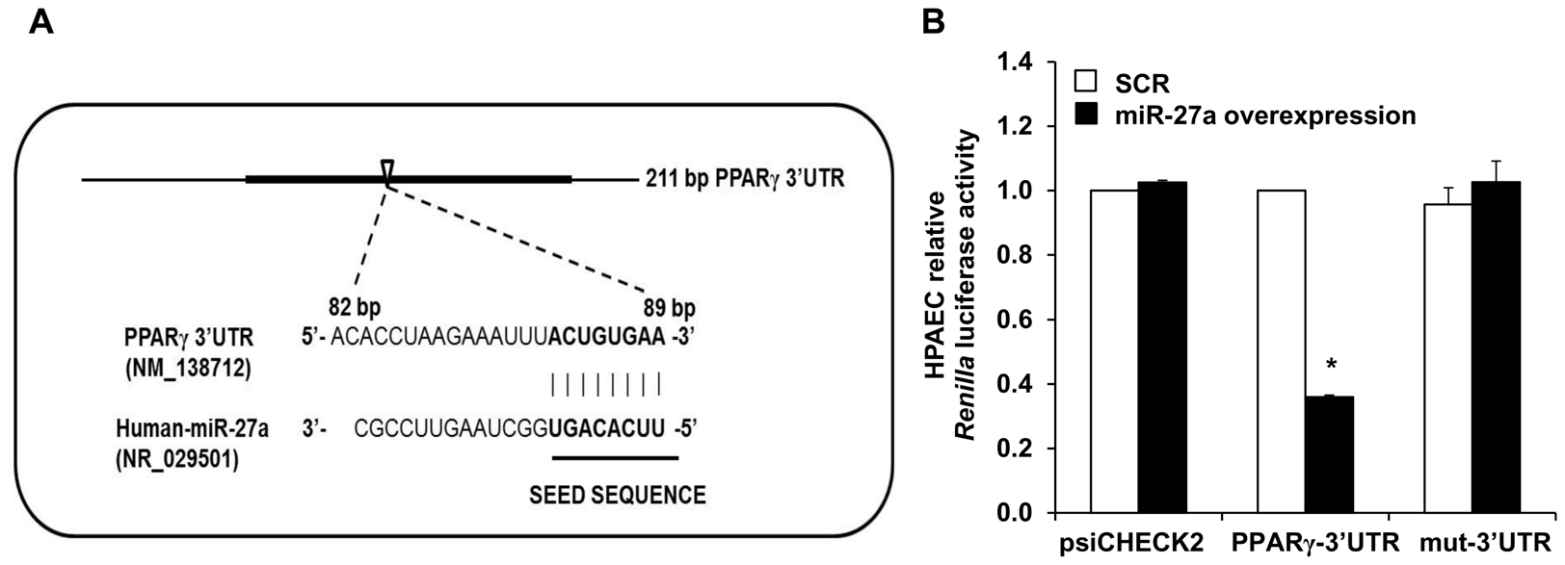
PPARγ is a target gene of miR-27a. **A.** Schematic illustration of the human PPARγ 3′UTR which contains a putative binding site (open arrowhead) for miR-27a. The miR-27a seed sequence is shown in bold font. **B.** HPAECs were incubated in a 24-well plate (1×10^4^ cells/well) for 24 h, washed with PBS, and then fresh growth medium was added before transfection with PPARγ-3′UTR luciferase reporter constructs. Wild-type vector (psiCHECK2) or psiCHECK2-PPARγ construct, or mut-3′UTR construct with or without 30 nM of miR-27a mimic or scrambled miRNA (SCR) were transiently co-transfected into HPAECs. After 72 h, HPAEC lysates were harvested and analyzed for *Renilla* and firefly luciferase activities. Each bar represents *Renilla* luciferase activity in relative light units normalized to firefly luciferase activity and expressed relative to HPAECs treated with SCR constructs. n = 3, * p<0.05 vs. SCR-PPARγ-3′UTR constructs.

To confirm that miR-27a binds directly to the 3′UTR of PPARγ (Figure 2A), we constructed a luciferase reporter DNA construct of the human 236 bp PPARγ mRNA 3′UTR containing the miR-27a binding site and used the psiCHECK2 vector to insert it into HPAECs. Figure 2A shows the predicted conserved binding sequence for miR-27a in the human PPARγ-3′UTR. As shown in Figure 2B, when miR-27a was co-transfected into HPAECs with psiCHECK2-PPARγ-3′UTR luciferase reporter construct, PPARγ luciferase activity of psiCHECK2-PPARγ-3′UTR was repressed by 65% compared with co-transfection with scrambled miRNA. In contrast, no changes in luciferase activity were observed in the psiCHECK2 wild-type reporter without PPARγ-3′UTR or psiCHECK2-PPARγ-3UTR^mut^ construct (mut-3′UTR) upon miR-27a overexpression or scrambled miRNA. These result clearly indicated that miR-27a directly binds to the PPARγ-3′UTR, and suppresses PPARγ expression.

### miR-27a Overexpression Stimulates HPAEC Proliferation and Reduces PPARγ Expression

**Figure 3:**
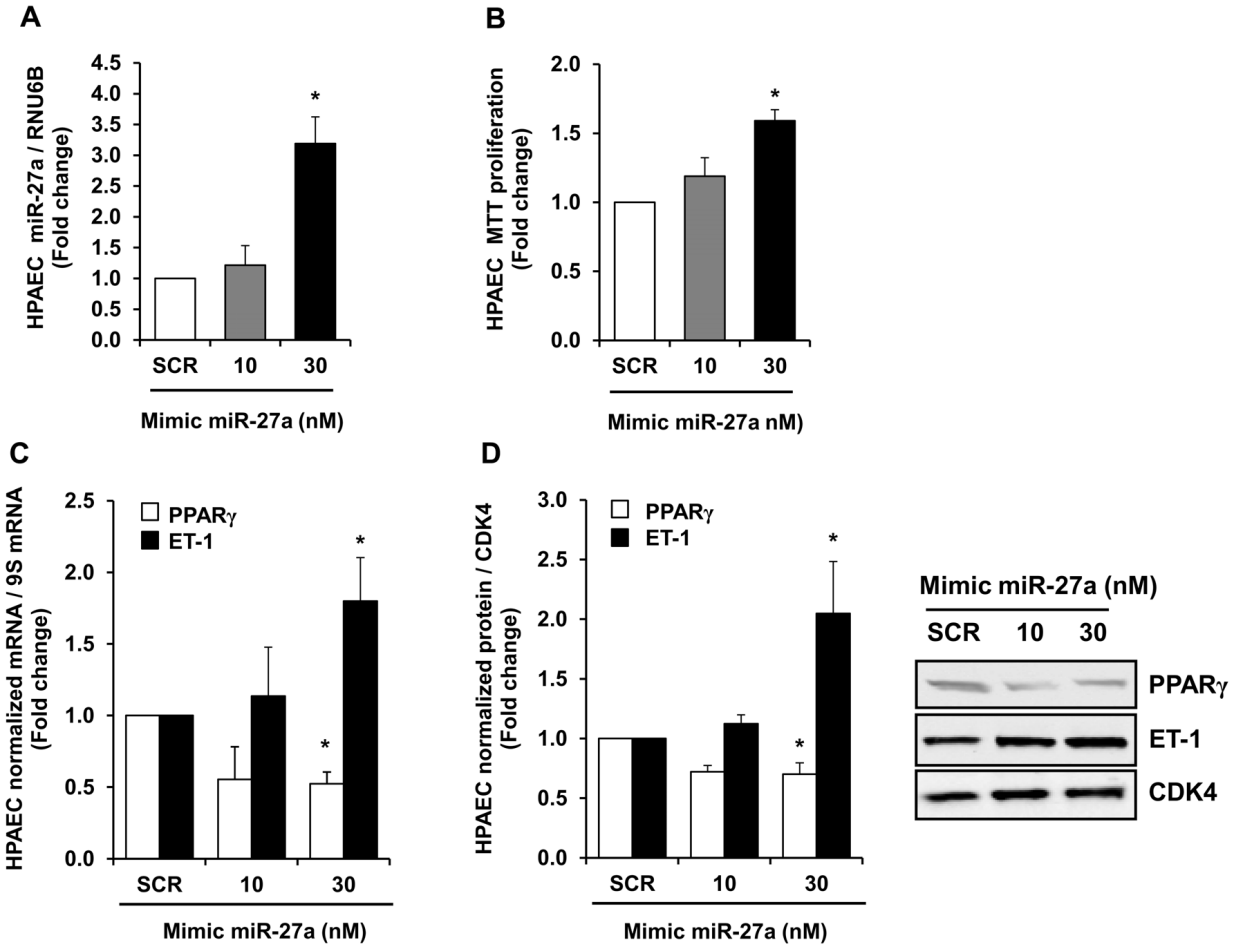
Overexpression of miR-27a with a miR-27a mimic increased miR-27a levels, HPAEC proliferation, and ET-1 levels and reduced PPARγ levels. HPAECs were exposed to normoxia (NOR) for 72 h and treated with graded concentrations of miR-27a mimic. qRT-PCR was employed to detect alterations in miR-27a, PPARγ, and ET-1 levels. Western blotting was employed to detect PPARγ and ET-1 protein levels. HPAEC proliferation was determined using MTT assays. Each bar represents the mean ± SE miR-27a (**A**), proliferation (**B**), or PPARγ or ET-1 mRNA relative to ribosomal S9 (9S RNA) (**C**), or PPARγ or ET-1 protein relative to CDK4 protein (**D**) expressed as fold-change vs. control. *p<0.05 vs. scrambled (SCR) miR mimic, n = 3–5.

In addition to increasing miR-27a as shown in Figure 1, hypoxia reduces PPARγ and stimulates HPAEC proliferation [10]. To confirm the impact of miR-27a on HPAEC PPARγ expression and proliferation, HPAEC were treated with graded concentrations of miR-27a mimic (10–30 nM). As shown in Figure 3, miR-27a mimic produced concentration-dependent increases in HPAEC miR-27a levels (Figure 3A) that were associated with increases in HPAEC proliferation detected by MTT assay (Figure 3B,) and ET-1 expression (Figures 3C and 3D) and reductions in PPARγ mRNA (Figure 3C) and protein (Figure 3D) levels. Reductions in PPARγ caused by 30 nM miR-27a mimic were comparable to those caused by hypoxia as previously reported [12], and 30 nM miR-27a mimic also caused maximal increases in HPAEC proliferation. These findings support previous evidence that miR-27a is upregulated under conditions associated with PH and establish that miR-27a reduces PPARγ expression.

### miR-27a Inhibition Attenuates HPAEC Proliferation and Increases PPARγ Expression

**Figure 4:**
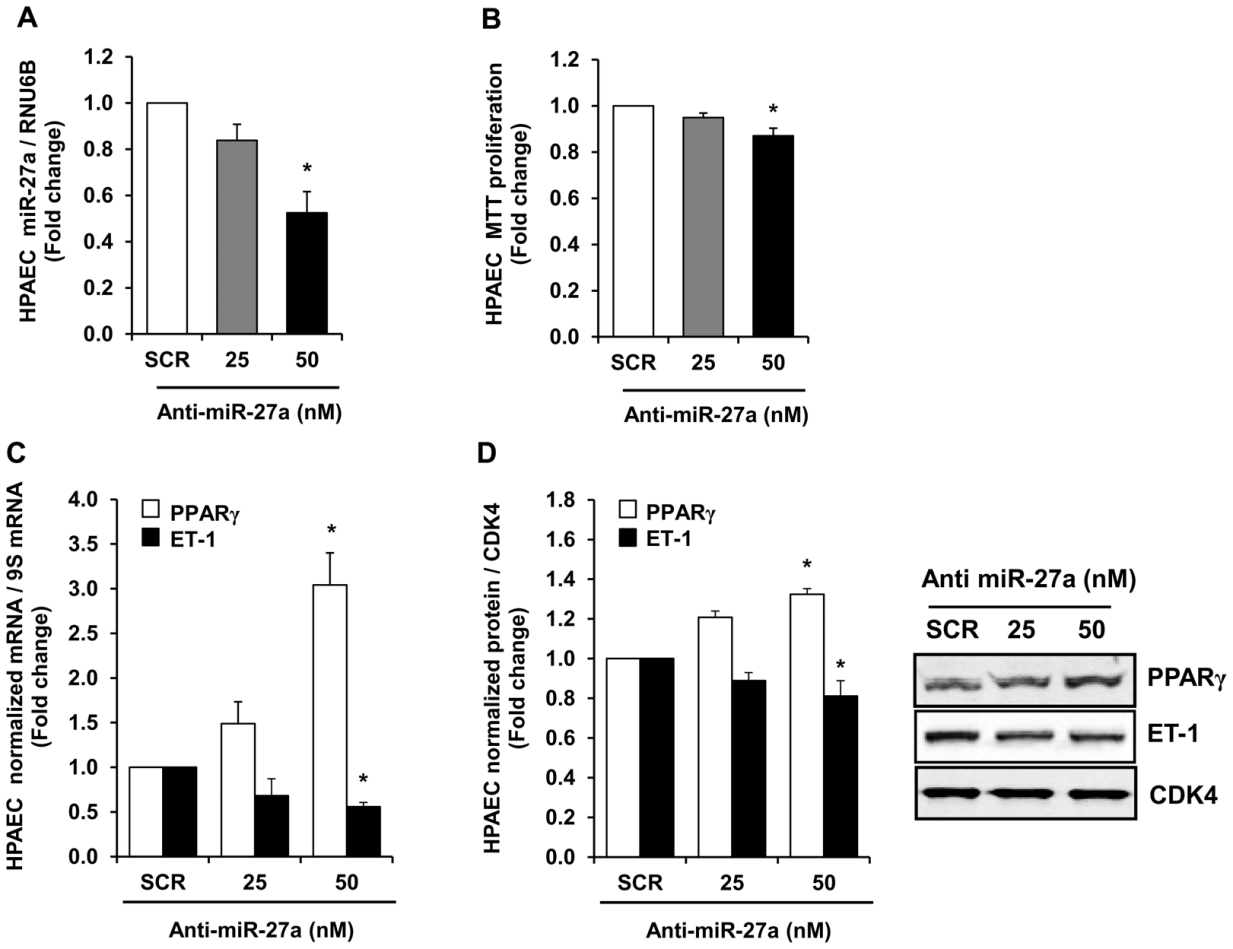
Anti-miR-27a reduced HPAEC miR-27a levels, HPAEC proliferation, and ET-1 levels and increased PPARγ expression. HPAECs were treated with either scrambled (SCR) or 25–50 nM anti-miR-27a for 72 hours. HPAEC were then collected, and RNA was isolated. qRT-PCR was performed for miR-27a, PPARγ, and ET-1 levels. Western blotting was employed to detect PPARγ and ET-1 protein levels. HPAEC proliferation was determined using MTT assays. Each bar represents the mean ± SE miR-27a/RNU6B (**A**), proliferation (**B**), PPARγ or ET-1 mRNA relative to ribosomal S9 (9S RNA) (**C**), or PPARγ or ET-1 protein relative to CDK4 protein (**D**) expressed as fold-change vs. SCR. *p<0.05 vs. scrambled (SCR) miRNA, n = 3–5.

To examine the effect of miR-27a inhibition on hypoxia-induced reductions in HPAEC PPARγ expression and proliferation, loss-of-miR-27a function was achieved by transfecting HPAECs with a miR-27a inhibitor. Whereas increased levels of miR-27a reduced PPARγ and stimulated HPAEC proliferation (Figure 3), miR-27a inhibitor had the opposite effect. As shown in Figure 4, treating HPAEC with graded concentrations of anti-miR-27a reduced miR-27a levels as expected (Figure 4A), attenuated basal HPAEC proliferation (Figure 4B) and decreased ET-1 expression (Figure 4C and 4D), and increased PPARγ mRNA (Figure 4C) and protein levels (Figures 4D). Taken together, the findings in Figures 3 and 4 provide compelling evidence that miR-27a regulates PPARγ expression and HPAEC proliferation.

### PPARγ Loss of Function Increases HPAEC Proliferation and ET-1 Protein Levels

**Figure 5:**
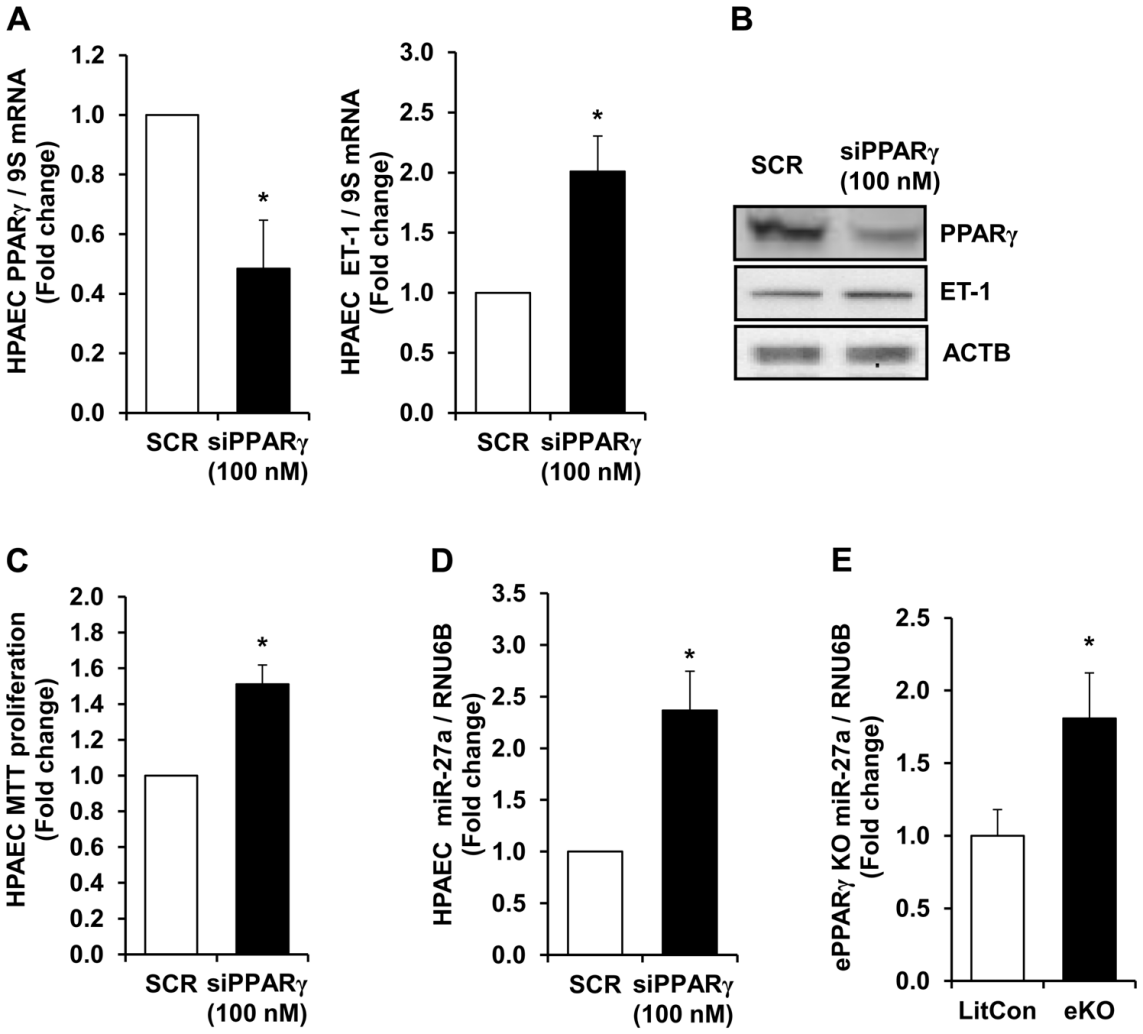
siRNA-mediated reductions in PPARγ increased HPAEC ET-1, proliferation, and miR-27a levels. HPAEC were treated with scrambled (SCR) or siRNA (100 nM) to PPARγ for 72 hours. HPAEC mRNA or proteins were then isolated. In selected studies, HPAEC were subjected to MTT assays of proliferation. **A.** qRT-PCR was performed demonstrating decreases in PPARγ mRNA levels following treatment with siRNA. Each bar represents the mean ± SE PPARγ or ET-1 relative to 9S in the same sample expressed as fold-change vs. cells treated with scrambled siRNA (SCR). n = 4–9, *p<0.05 vs. SCR. **B.** HPAECs were treated with 100 nM SCR or PPARγ siRNA, and proteins were subjected to Western blotting for PPARγ, ET-1, or β-actin (ACTB). As illustrated by these representative Western blots, PPARγ depletion reduced HPAEC PPARγ protein levels, and increased levels of ET-1. **C.** HPAEC were treated with 100 nM SCR or siPPARγ and subjected to MTT proliferation assays. n = 6. *p<0.05 vs. SCR. **D.** HPAECs were treated with SCR or siPPARγ, and miRNA was isolated and subjected to qRT-PCR analysis along with RNU6B. Each bar represents the mean ± SE miR-27a relative to RNU6B expressed as fold changed vs. SCR. n = 4, *p<0.05 vs. SCR. **E.** miR-27a levels were upregulated in lungs from endothelial-targeted PPARγ knockout (ePPARγ KO) mice. miR-27a levels in lungs from endothelial-targeted PPARγ KO mice were determined with qRT-PCR. Each bar represents the mean ± SE level of miR-27a relative to RNU6B expressed as fold-change vs. littermate controls (LitCon), n = 3–6. *p<0.05 vs. LitCon.

The findings in Figures 3 and 4 indicate that alterations in miR-27a levels are sufficient to regulate PPARγ levels and HPAEC proliferation. To further determine whether alterations in PPARγ expression were sufficient to increase HPAEC proliferation, siRNA to PPARγ was employed to directly and selectively reduce HPAEC PPARγ levels followed by examination of proliferative mediator ET-1 that we have previously reported to be regulated by PPARγ as well as HPAEC proliferation. Figure 5A illustrates that siRNA-mediated knockdown of HPAEC PPARγ to levels comparable to those observed in hypoxia-exposed HPAEC increased ET-1 mRNA levels. The findings in Figure 5B illustrate that PPARγ siRNA effectively reduced PPARγ protein and increased ET-1 protein. ACTB was included to demonstrate equal protein loading between the lanes. Figure 5C demonstrates that these reductions in PPARγ are sufficient to promote HPAEC proliferation. To further examine the association between PPARγ and miR-27a, levels of miR-27a in siPPARγ HPAECs were examined. Reductions in PPARγ significantly increased miR-27a levels (Figure 5D). As illustrated in Figure 5E, compared to lungs from littermate control mice, miR-27a levels were upregulated in lungs from endothelial-targeted PPARγ knockout (ePPARγ KO) mice [39].

### PPARγ Activation Attenuates Increases in miR-27a Levels in Hypoxia-exposed Mouse Lung or HPAECs

**Figure 6:**
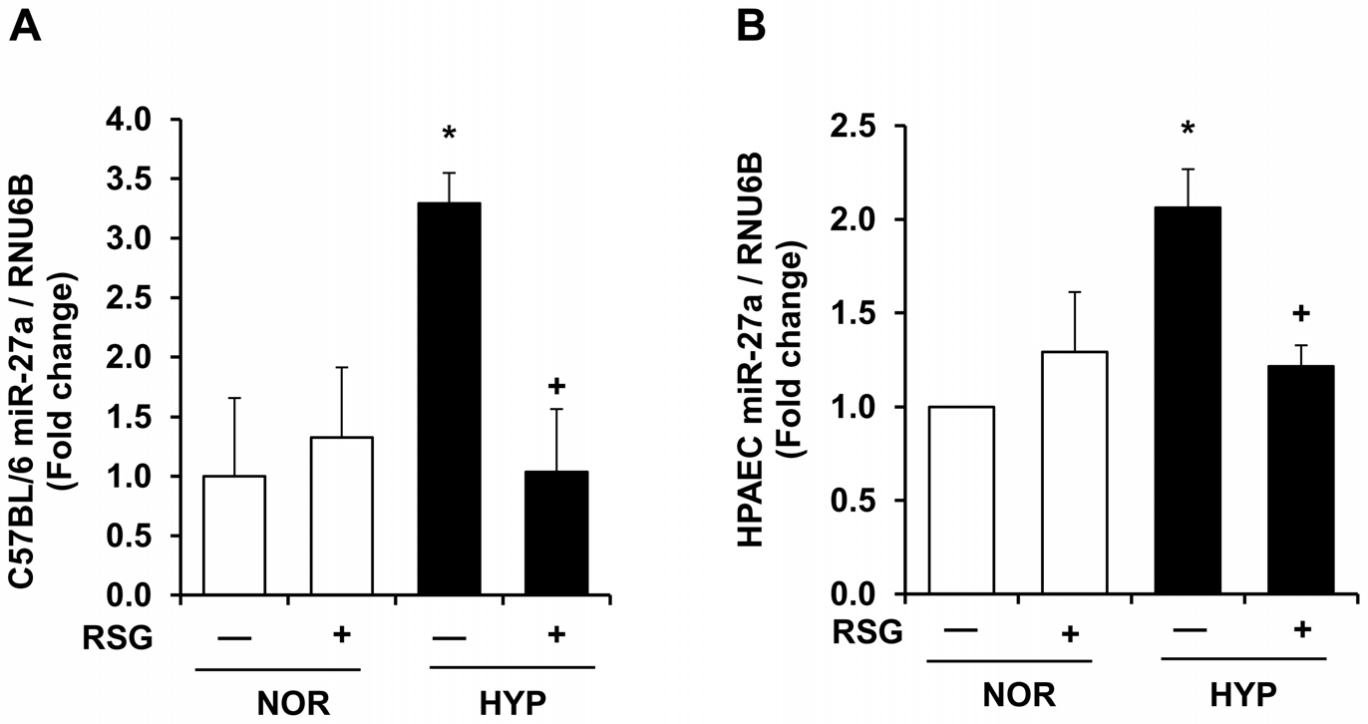
A PPARγ ligand attenuates hypoxic increases in miR-27a levels in mouse lung or in HPAECs. **A.** Whole lung homogenates were collected from mice exposed to normoxia (NOR, 21% O_2_) or hypoxia (HYP, 10% O_2_) for 3-weeks. During the last 10-days of this exposure, selected animals were also treated ± rosiglitazone (RSG, 10 mg/kg/d by gavage) as we have reported. [12] Lung miR-27a levels were measured with qRT-PCR and are expressed relative to lung RNU6B. *p<0.05 vs. NOR, ^+^p<0.05 vs. HYP, n = 3–4. **B.** HPAECs were exposed to NOR (21% O_2_) or HYP (1% O_2_) for 72 h**.** Selected cells were treated during the final 24 h of exposure with RSG (10 µM). Each bar represents the mean miR-27a level relative to RNU6B ± SE. *p<0.05 vs. NOR, ^+^p<0.05 vs. HYP, n = 3–6.

While the findings in Figures 3 and 4 demonstrate that miR-27a regulates PPARγ, the results in Figure 5 suggest that PPARγ reciprocally regulates miR-27a levels. To confirm these relationships, samples were examined from PH models including hypoxia-exposed mice [12] and hypoxia-induced HPAEC proliferation in vitro [10]. Figure 6A illustrates lung miR-27a levels in mice exposed to hypoxia (10% O_2_) for 3-weeks ± treatment with the PPARγ ligand, RSG, during the final 10-days of exposure. In this model, these conditions caused PH, right ventricular hypertrophy, and muscularization of small pulmonary arteries, and treatment with RSG attenuated these hypoxia-induced derangements [12]. Hypoxia also caused significant increases in lung miR-27a levels that were attenuated by treatment with RSG (Figure 6A). Similarly in HPAEC exposed to control or hypoxic (1% O_2_) conditions for 72 hours ± treatment with RSG (10 µM) during the last 24 hours, PPARγ activation attenuated hypoxia-induced ET-1 expression and proliferation [10]. The findings in Figure 6B demonstrate that hypoxia also increased HPAEC miR-27a levels in vitro and that treatment with RSG attenuated increases in miR-27a in hypoxia-exposed cells.

### Knockdown of Transcription Factors, SP1 and EGR1, Attenuates miR-27a Expression

**Figure 7:**
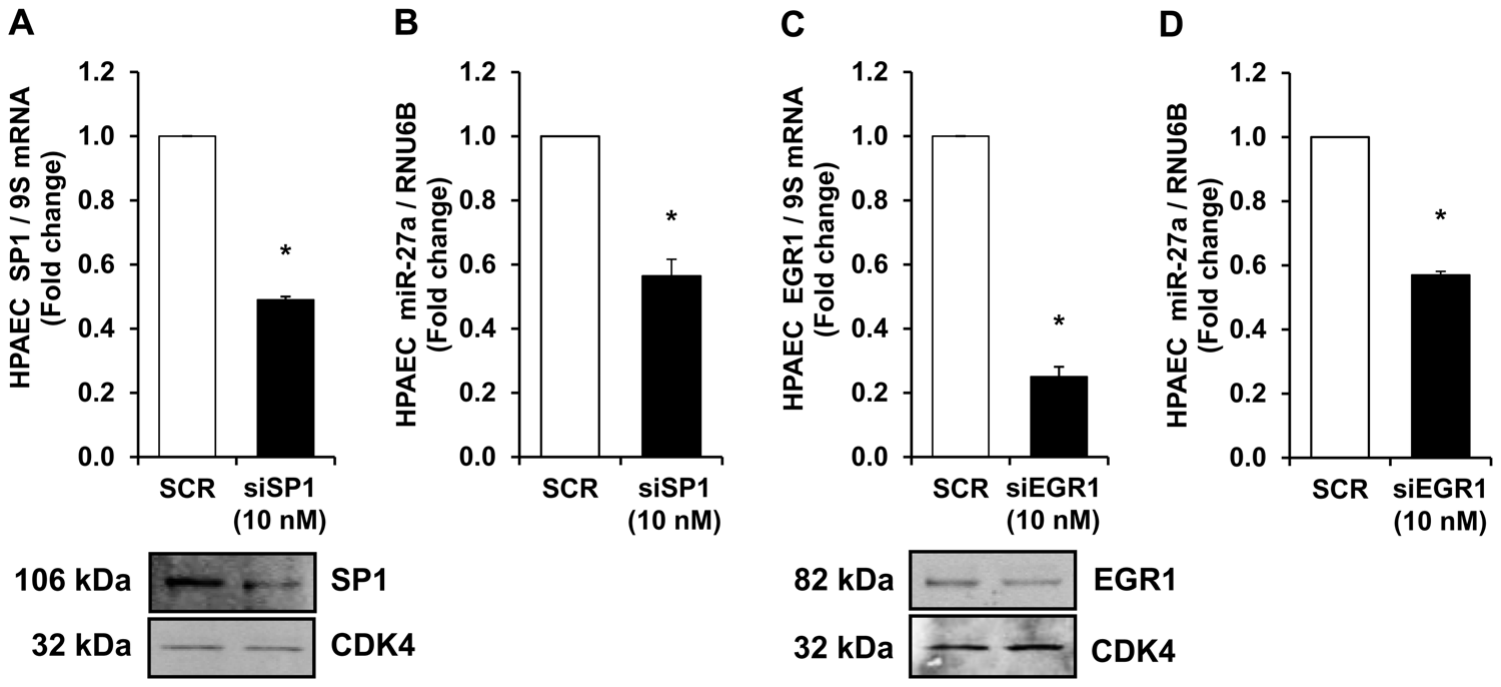
Knockdown of transcription factors, SP1 and EGR1, attenuates miR-27a expression. HPAEC were treated with 10(SCR) or siRNA duplexes to SP1 or EGR1 for 72 hours. HPAEC mRNA or proteins were then isolated. qRT-PCR was performed demonstrating decreases in SP1 (**A**) or EGR1 (**C**) mRNA levels following treatment with siRNA. Each bar represents the mean ± SE SP1 or EGR1 relative to 9S in the same sample expressed as fold-change vs. cells treated with scrambled siRNA (SCR). n = 3, *p<0.05 vs. SCR. Western blotting was employed to detect SP1 (**A**) or EGR1 (**C**) protein levels. Representative blots depicting SP1 or EGR1 protein depletion in siRNA-treated HPAEC is shown. n = 3, *p<0.05 vs. SCR. HPAECs were treated with SCR or siRNA duplexes to SP1 or EGR1, and miRNA was isolated and subjected to qRT-PCR analysis along with RNU6B. Each bar represents the mean ± SE miR-27a relative to RNU6B expressed as fold change vs. SCR. n = 3, *p<0.05 vs. SCR. miR-27a levels were downregulated following SP1 (**B**) or EGR1 (**D**) depletion in HPAECs.

Transcription factors such as SP1 [40] and EGR1 [41] have been reported to stimulate miR-27a expression. Because our preliminary data demonstrate that hypoxia increased SP1 and EGR1 expression in mouse lungs (not shown), RNAi duplexes to SP1 (10 nM) or EGR1 (10 nM) were employed to directly reduce HPAEC SP1 or EGR1 levels. Figure 7A and 7C illustrate that knockdown of HPAEC SP1 or EGR1 decreased SP1 or EGR1 mRNA and protein levels, respectively. The findings in Figure 7B and 7D illustrate that depletion of either SP1 or EGR1 was sufficient to significantly attenuate miR-27a expression. Taken together, these findings provide compelling evidence that SP1 or EGR1 regulate miR-27a expression in HPAECs.

## Discussion

Pulmonary hypertension is a serious cardiopulmonary disorder defined by increases in pulmonary artery pressure and pulmonary vascular resistance that cause significant morbidity and mortality [1]–[3]. Although existing PH therapies are designed to attenuate these derangements, the poor outcomes in PH [2], [4]–[6] highlight an urgent need for a more complete understanding of the complex pathobiology that causes PH and for novel therapeutic strategies. Chronic hypoxia-induced PH is a frequent clinical problem that contributes to endothelial dysfunction, pulmonary vasoconstriction, pulmonary vascular remodeling, right ventricular hypertrophy, and death. We have recently reported that hypoxia activates transcriptional mechanisms involved in upregulating multiple components of the ET-1 signaling pathway and that activating the PPARγ receptor attenuates hypoxia-induced PH and ET-1 expression in mouse lungs and HPAECs [10]. Furthermore, hypoxia reduced PPARγ expression in the mouse lung and pulmonary vascular wall cells through an ERK-NF-κB-NOX4-dependent pathway [21]. The current results clarify that hypoxia reduces PPARγ through post-transcriptional pathways involving miR-27a that increase endothelial ET-1 expression and proliferation. Additionally, this study provides novel evidence that activation of the PPARγ receptor attenuates hypoxia-induced miR-27a expression. Collectively, these studies demonstrate that miR-27a and PPARγ mediate mutually repressive actions in hypoxic pulmonary vasculature and that targeting PPARγ may represent a novel therapeutic approach in PH.

Rapidly emerging evidence supports the role of miRNAs in PH pathogenesis [22]–[26], [28]–[33], [42]–[44], although the full range of targets regulated by miRNA remains to be established. For example, Caruso and coworkers used high-throughput miRNA array analysis to detect hypoxia-induced miRNAs (so-called hypoxamirs) caused by chronic hypoxia in rats [22]. In human and animal subjects, hypoxia induces the expression of a number of miRNAs [38] (miR-17, miR-21, miR-138, miR-143/145, miR-204, miR-206, miR-210, and miR-424) that contribute to PH pathogenesis [22], [24]–[26], [29], [44]–[47]. In PASMCs, hypoxia increased miR-21 [25], [48] and miR-210 [45] and reduced miR-206 [46] to increase proliferation and migration. In contrast, although ECs play a pivotal role in pulmonary vascular biology, less is known about the role of miRNAs in the alteration of PAEC function.

The current study focused on miR-27 because it: 1) regulates PPARγ, 2) is highly expressed in the lung and heart [49]–[51], 3) is increased in the lungs of animals with PH [22] and in PASMC isolated from patients with IPAH [30], and 4) participates in the regulation of proliferation and differentiation in multiple cell types [23], [35], [45], [52]–[56]. Because PPARγ was reduced in hypoxia-induced PH [11], [12], we hypothesized that hypoxia would increase PPARγ-related miRNAs to reduce PPARγ expression leading to increased ET-1 expression and PH. Because the PPARγ 3′UTR contains putative binding sites for miR-27a/b, -130a/b, -301a/b, and -454, we measured levels of these miRNAs in the lungs from hypoxia-exposed mice and in HPAECs in vitro. Hypoxia upregulated miR-27a and -27b levels in HPAECs, whereas only miR-27a was increased in mouse lungs. In contrast, hypoxia downregulated levels of other miRNAs predicted to regulate PPARγ (Figure 1). Although the pattern of hypoxia-induced alterations in the expression of these miRNAs was similar in mouse lung and HPAECs, we speculate that hypoxia-induced changes in miRNA levels in lung homogenates were less pronounced due to the contribution of multiple cell types to whole lung miRNA analysis. Differences between in vivo and in vitro models, species, or degree of hypoxia may have contributed as well. Nonetheless, because miR-27a was increased by hypoxia in both in vivo and in vitro models, we focused on increased miR-27a levels as a putative mechanism for reductions in PPARγ and confirmed with reporter assays that miR-27a binds to the PPARγ 3′UTR in HPAECs. Furthermore, our studies demonstrate that miR-27a mimic reduced PPARγ, increased ET-1, and stimulated HPAEC proliferation whereas anti-miR-27a increased PPARγ and reduced ET-1 and HPAEC proliferation. The proximate role of reductions in PPARγ was confirmed by in vitro studies using siPPARγ. siRNA-mediated PPARγ knockdown was sufficient to increase HPAEC ET-1 levels and increase basal HPAEC proliferation.

**Figure 8:**
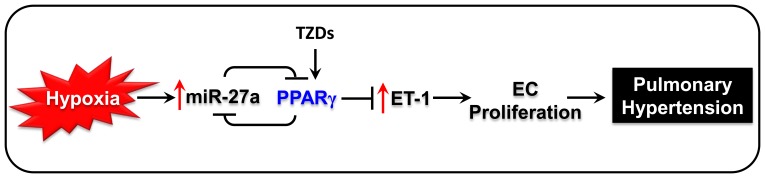
Putative pathways by which PPARγ and miR-27a regulate ET-1 in PH. The current findings indicate that hypoxia increases lungs miR-27a expression and that increases in miR-27a reduce PPARγ levels which stimulates increased ET-1 levels and pulmonary vascular cell proliferation. Conversely, activating PPARγ with rosiglitazone attenuated hypoxic upregulation of miR-27a and ET-1. These findings suggest that PPARγ ligands attenuate alterations in miR-27a and ET-1 levels to reduce PH.

Because these findings demonstrated that hypoxia increased miR-27a and reduced PPARγ in vitro and in vivo and because PPARγ ligands restored hypoxia-induced reductions in PPARγ [11], we examined the ability of PPARγ activation with pharmacological ligands to attenuate hypoxic increases in miR-27a levels. Consistent with evidence that activation of PPARγ with rosiglitazone attenuates hypoxic reductions in PPARγ in rat lung, rosiglitazone treatment attenuated hypoxia-induced increases in miR-27a in mouse lung and in HPAEC (Figure 6). These results provide novel evidence that PPARγ activation suppresses hypoxic increases in miR-27a. Taken together our findings provide evidence for a mutually repressive relationship between miR-27a and PPARγ. As illustrated in Figure 8, these findings suggest a pathogenic cascade wherein hypoxia-induced increases in miR-27a reduce PPARγ and increase ET-1 to stimulate HPAEC proliferation. However, stimulating the activity of remaining levels of PPARγ with pharmacological ligands can reduce miR-27a (Figure 6). We previously reported that these identical PPARγ ligand treatment conditions attenuate ET-1, PH, and vascular remodeling in vivo [12] and ET-1 and HPAEC proliferation in vitro [10]. Taken together these results define previously undescribed roles for and regulation of PPARγ in hypoxic pulmonary vascular cells.

These studies have several important limitations. First, hypoxic mouse models fail to completely recapitulate the pathological changes seen in the pulmonary vasculature of patients with pulmonary arterial hypertension (PAH) [57]. However observations that PPARγ is reduced [7] and ET-1 is increased [19], [58] in the lungs and pulmonary vascular tissue of patients with PAH suggests that the biology observed in the hypoxic mouse model has relevance to human disease. Secondly, our study does not directly address how hypoxia increases miR-27a levels or how PPARγ activity suppresses this effect. Transcription factors such as SP1 [40] and EGR1 [41] promote miR-27a expression, whereas PPARγ activation inhibits SP1 [59] and EGR1 [60] in selected systems. Our results demonstrate that knockdown of SP1 or EGR1attenuated miR-27a levels in HPAECs. These findings suggest that PPARγ activation could lead to transrepression of hypoxia-activated transcription factors that activate the miR-27a promoter. Further studies will be required to fully elucidate the molecular mechanisms by which hypoxia increases miR-27 expression and if preventing increases in miR-27a levels is sufficient to prevent or reverse hypoxic-induced ET-1 expression and PH in vivo.

To our knowledge, the current report provides the first evidence that hypoxia inhibits PPARγ expression and increases ET-1 expression and HPAEC proliferation through miR-27a-mediated post-transcriptional mechanisms in vivo and in vitro. Furthermore, these studies provide novel evidence for a previously unrecognized mutually repressive relationship between PPARγ and miR-27a in hypoxic pulmonary vasculature. These results suggest that targeting PPARγ may represent a novel therapeutic approach in PH.
